# Evolutionary History of Oxysterol-Binding Proteins Reveals Complex History of Duplication and Loss in Animals and Fungi

**DOI:** 10.1177/25152564221150428

**Published:** 2023-01-11

**Authors:** Rohan P. Singh, Yu-Ping Poh, Savar D. Sinha, Jeremy G. Wideman

**Affiliations:** Center for Mechanisms of Evolution, Biodesign Institute, School of Life Sciences, 7864 Arizona State University, Tempe, USA

**Keywords:** oxysterol-binding proteins, lipid transport proteins, phylogenetics, membrane contact sites, evolutionary cell biology

## Abstract

Cells maintain the specific lipid composition of distinct organelles by vesicular transport as well as non-vesicular lipid trafficking via lipid transport proteins. Oxysterol-binding proteins (OSBPs) are a family of lipid transport proteins that transfer lipids at various membrane contact sites (MCSs). OSBPs have been extensively investigated in human and yeast cells where 12 have been identified in *Homo sapiens* and 7 in *Saccharomyces cerevisiae*. The evolutionary relationship between these well-characterized OSBPs is still unclear. By reconstructing phylogenies of eukaryote OSBPs, we show that the ancestral Saccharomycotina had four OSBPs, the ancestral fungus had five OSBPs, and the ancestral animal had six OSBPs, whereas the shared ancestor of animals and fungi as well as the ancestral eukaryote had only three OSBPs. Our analyses identified three undescribed ancient OSBP orthologues, one fungal OSBP (Osh8) lost in the lineage leading to yeast, one animal OSBP (ORP12) lost in the lineage leading to vertebrates, and one eukaryotic OSBP (OshEu) lost in both the animal and fungal lineages.

## Introduction

Eukaryotes are characterized by the intricate intracellular membrane-bound organelles of the endomembrane system like the endoplasmic reticulum (ER), the Golgi, endosomes, lysosomes, peroxisomes, and plasma membrane (Lippincott-Schwartz & Phair, 2010). While trafficking of cellular components via vesicular transport between these organelles is well studied, how different organelles maintain specific lipid composition is less clear. Most lipids are synthesized in the ER and then transported to other cellular compartments in vesicles (Fagone & Jackowski, 2009). However, some organelles like mitochondria and chloroplasts are not part of the endomembrane system and require non-vesicular lipid transport to obtain their lipids (Flis & Daum, 2013). To maintain appropriate organelle-specific lipid ratios, even organelles that partake in vesicular transport require non-vesicular lipid transport (Holthuis & Levine, 2005; Levine, 2004). Recent advances demonstrate that non-vesicular lipid transport largely occurs at points of close contact between organelles called membrane contact sites (MCSs) (Prinz et al., 2020). MCSs are present between the ER and nearly every other membrane-bound organelle where they promote interaction and communication between organelles and facilitate lipid exchange by lipid transport proteins (LTPs) (Delfosse et al., 2020; Prinz et al., 2020).

OSBPs are an important family of LTPs in eukaryotes; however, their functional significance is elusive as only any one of seven yeast OSBPs can complement the absence of the other six (Beh et al., 2001; Tong et al., 2021). An elegant hypothesis for how OSBPs contribute to organellar phospholipid homeostasis was proposed by Tong et al. (2016). Although OSBPs are generally thought of as lipid exchangers, Tong et al. (2016) suggest that the primary function of OSBPs is the recycling of phosphatidylinositol 4-phosphate (PI4P) from the plasma membrane to the ER, where it is immediately converted into phosphatidylinositol (PI). Indeed, the binding and transport of PI4P have been recently shown as the only essential function of OSBPs in yeast (Tong et al., 2021). The universality of PI4P binding by OSBPs is conferred by the lipid binding motif EQVSHHPP of the OSBP-Related Domain (ORD), which is present in all known OSBPs (de Saint-Jean et al., 2011; Tong et al., 2013). Slight changes in the structure of the lipid-binding site of OSBPs enable binding of a secondary ligand like sterol or phosphatidylserine (PS) (Delfosse et al., 2020; Nakatsu & Kawasaki, 2021; Raychaudhuri & Prinz, 2010; Tong et al., 2016; Moser von Filseck et al., 2015). In these cases, OSBPs use the PI4P gradient (PM > Golgi > ER) to move substrates including sterols and PS (Ridgway et al., 1992; Tong et al., 2016). Several OSBPs contain a Pleckstrin Homology (PH) Domain (Lemmon, 2008), which mediates membrane and phosphoinositide recognition and association enabling different OSBPs to specifically localize to different membranes (Di Paolo & De Camilli, 2006). Thus, the constant flow of PI4P from the PM to the ER where it is converted to PI drives the transport of PS and cholesterol to other membranes, even against their concentration gradients (e.g., Moser von Filseck et al., 2015), thereby contributing to the maintenance of organellar identity.

In addition to the ORDs and PH domains, OSBPs can be decorated by other functional domains. For example, OSBPs often contain an FFAT (two phenylalanines followed by an acidic tract) motif recognized by ER-localized VAMP-Associated Protein (VAP or Scs2 in yeast) (Loewen et al., 2003), which enables OSBPs to tether the ER and transfer lipids between various organelles (Kamemura & Chihara, 2019). Other OSBPs have Ankyrin Repeat Domains (ARD; Delfosse et al., 2020) that putatively enable protein-protein interactions (e.g., Johansson et al., 2005; Tong et al., 2019). These additional domains hone and sculpt the biochemical capacities of various OSBPs.

With 12 OSBPs in *Homo sapiens* and 7 in *Saccharomyces cerevisiae*, alongside reports indicating a large degree of redundancy (e.g., Tong et al., 2021), individuating OSBP function has been extremely challenging. Phylogenetic analyses of highly paralogous protein families can provide clarification of relationships between proteins in different organisms as well as functional insight (Hellmuth et al., 2015). Here, we present a phylogenetic analysis of eukaryotic OSBPs partially resolving the relationship between OSBPs in opisthokonts, the lineage that includes animals, fungi, and their closest protist relatives.

## Methods

To determine how OSBPs in *H. sapiens* and *S. cerevisiae* are related, we used a phylogenetic approach. The 12 *H. sapiens* and 7 *S. cerevisiae* protein sequences were collected from National Center for Biotechnology Information (NCBI) and used as BLAST (Altschul et al., 1997) queries against predicted proteomes of diverse Eukaryotes using a subset of EukProt (Richter et al., 2022) proteomes as well as the Saccharomycotina database on Mycocosm at the Joint Genome Institute (JGI) web server (https://mycocosm.jgi.doe.gov/mycocosm/home; Grigoriev et al., 2014; Supplementary File S1). OSBP protein sequences were aligned using MUSCLE version 3.8 (Edgar, 2004). Protein alignments were trimmed using trimAL version 1.2 (Capella-Gutiérrez et al., 2009) to remove poorly aligned regions. The final sequence matrices were inspected in Mesquite version 3.70 (Maddison & Maddison, 2021) and manually trimmed as necessary. To help limit artifacts like long branch attraction, protein sequences >60% missing data and extremely long branches were removed from the analysis. Some sequences from the same species that branched sister to one another were also removed from the analysis to simplify the data matrix (e.g., 6 of 12 *Arabidopsis* OSBPs were removed). The phylogenetic reconstructions were inspected to determine likely orthology relationships. Five clade-specific data matrices were generated (Saccharomycotina, Holomycota, Holozoa, Opisthokonts, and Eukaryota) and the phylogenetic trees were inferred with IQTree version 2.0.3 (Minh et al., 2020) using the LG4X model. A few divergent sequences branched near the base of the tree with low support and were removed in final analyses.

## Results and Discussion

### The Ancestor of Saccharomycotina had Four Osh Proteins

The *S. cerevisiae* genome encodes seven OSBPs that aid in the transport of lipids throughout the cell, three pairs of which originated from the whole genome duplication in this clade (Marcet-Houben & Gabaldón, 2015). Thus, four classes of OSBPs have been delineated in *S. cerevisiae* (Beh et al., 2001). Osh1 and Osh2 both contain Ankyrin Repeat Domains, an ORD, and a PH domain and function similarly (Lehto et al., 2001; Loewen et al., 2003). Osh3 lacks a paralogue, contains a GOLD domain, a PH domain, a FFAT motif, and an ORD (Loewen et al., 2003). Osh4 and Osh5 are closely related and contain only an ORD (Raychaudhuri & Prinz, 2010). Similarly, Osh6 and Osh7 are closely related and contain only an ORD (Wong et al., 2021).

To determine if the four classes of OSBPs in *S. cerevisiae* are representative of Saccharomycotina, we reconstructed the phylogeny of Saccharomycotina Osh proteins (Figure S1). A total of 239 sequences of Osh proteins were collected aligned, trimmed, and subjected to phylogenetic analysis using IQTREE (Minh et al., 2020). As expected, three pairs derived from the whole genome duplication/fusion event (Marcet-Houben & Gabaldón, 2015) grouped with related proteins (see Figure S1). Apart from the lineage derived from the whole genome duplication, most Saccharomycotina species contained only four Osh proteins (some only contained three), each grouping within separate nearly fully supported ancestral Osh clades, which we have labeled Osh1/2, Osh3, Osh4/5, and Osh6/7. Thus, we infer that the ancestor of Saccharomycotina possessed four Osh proteins which likely functioned similarly to the protein pairs in *S. cerevisiae*. The retention of the expanded OSBP repertoire of *S. cerevisiae* and closely related yeasts could be explained by subfunctionalization (Force et al., 1999; Lynch & Force, 2000; Stoltzfus, 1999), whereby the combined function of the duplicate pairs was sufficiently performed by the ancestral pre-duplicate.

### The Ancestor of Fungi had Five Osh Proteins

**Figure 1.**
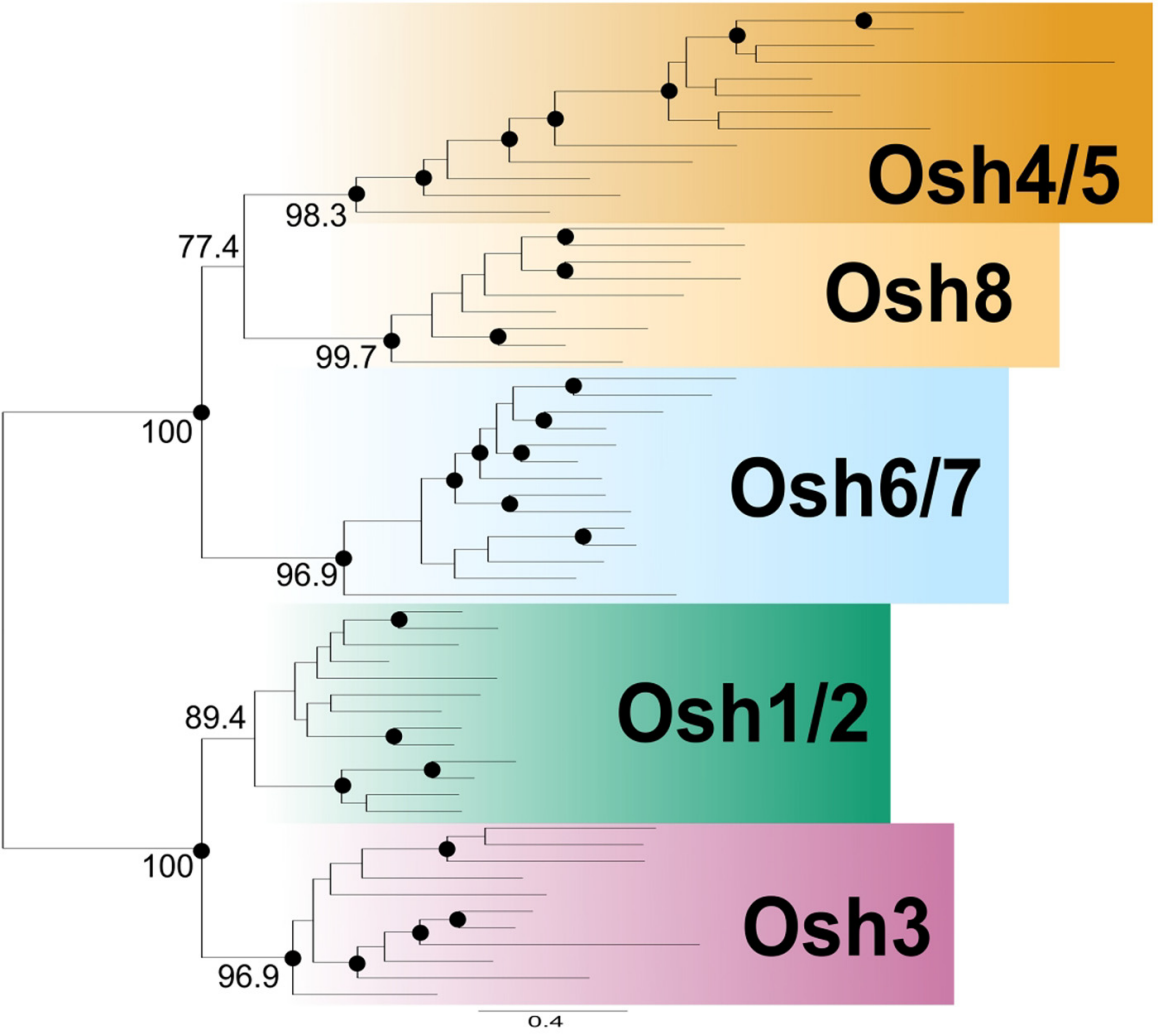
Five OSBPs were present in the ancestor of Holomycota. This schematic tree is based on Figure S2. Sequences were collected from EukProt and NCBI. The tree indicates that there were five OSBPs found in the last common ancestors of Holomycota—Osh1/2, Osh3, Osh4/5, Osh6/7, and Osh8. Nodes with the bootstrap values greater than 90 are shown in a solid circle (•), and the bootstrap values that supported the grouping of the five major clades are shown on the branches leading to the node.

To determine if the ancestral fungus had the same set of Osh proteins as the ancestor of Saccharomycotina, we extended our phylogenetic analysis to include representatives from every major holomycotan group (Figures 1 and S2). Surprisingly, the resulting phylogenetic reconstruction contained five Osh clades, four of which reflected the clades seen in the Saccharomycotina (Osh1/2, Osh3, Osh4/5, and Osh6/7) in addition to an unknown ancestral clade—which we name Osh8—lacking a representative *S. cerevisiae* Osh protein. Osh8 branches sister to Osh4/5 with modest support (Figure 1). Like Osh4/5, domain analysis of Osh8 revealed that it lacks both an FFAT motif and a PH domain and contains only an ORD. Thus, the ancestral fungus contained five Osh proteins, one of which (Osh8) was lost in the lineage leading to Saccharomycotina. These data reveal that a complex lipid transport system with five OSBPs was in place in the ancestral holomycotan. While the function of Osh8 is unclear, since it branches sister to Osh4/5, perhaps the functions of Osh4/5 and Osh8 are similar.

### The Ancestor of Animals had Six ORPs

**Figure 2.**
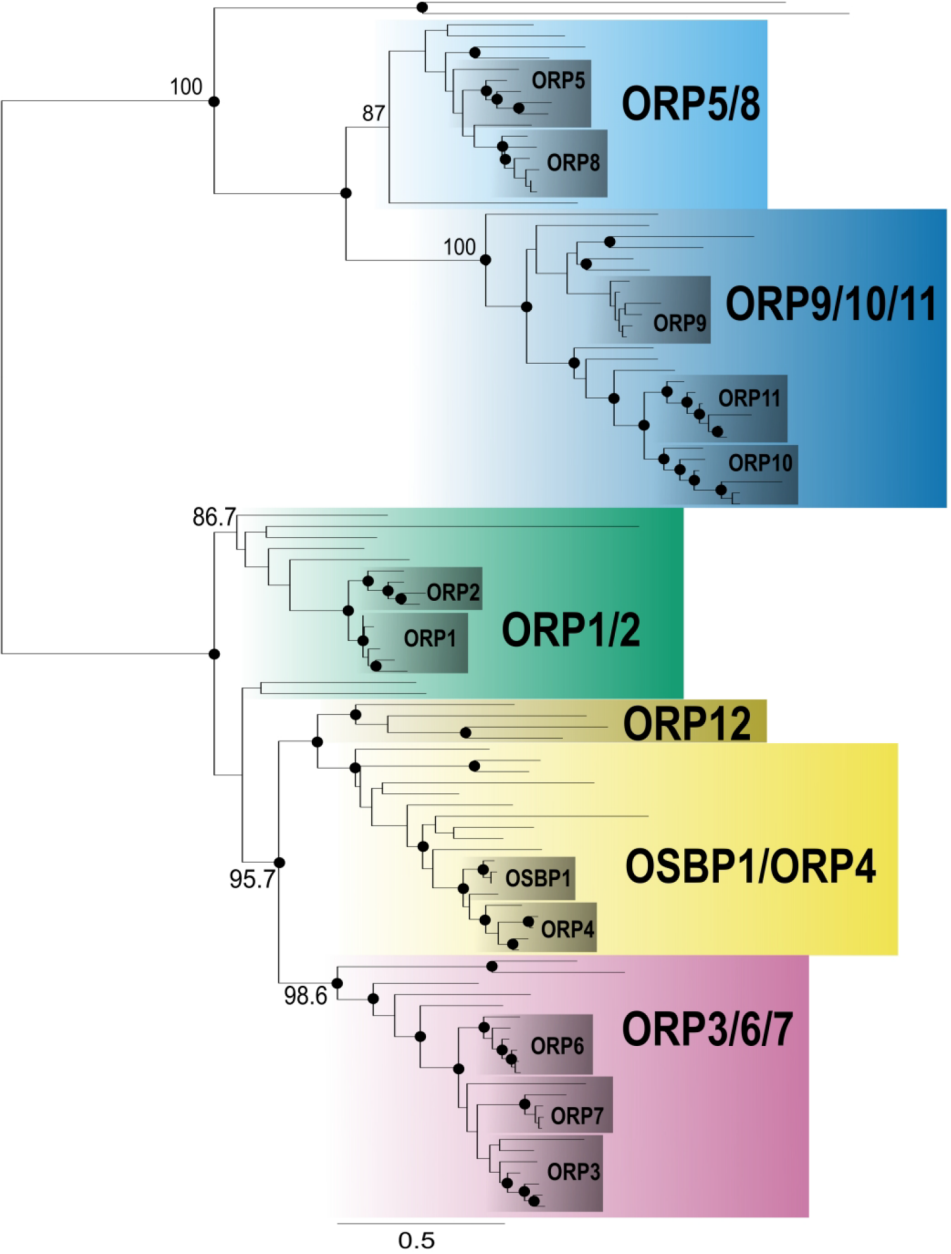
Six OSBPs were present in the ancestor of Holozoa. This schematic tree is based on Figure S3. Sequences were collected from EukProt and NCBI and included animals and their single-celled relatives. Six ancestral clades were identified (ORP1/2, OSBP1/ORP4, ORP3/6/7, ORP5/8, ORP9/10/11, and ORP12). The grey boxes highlight all the orthologues within the vertebrates. Nodes with the bootstrap values greater than 90 are shown in a solid circle (•), and the bootstrap values that supported the grouping of the six major clades are shown on the branches leading to the node.

**Figure 3.**
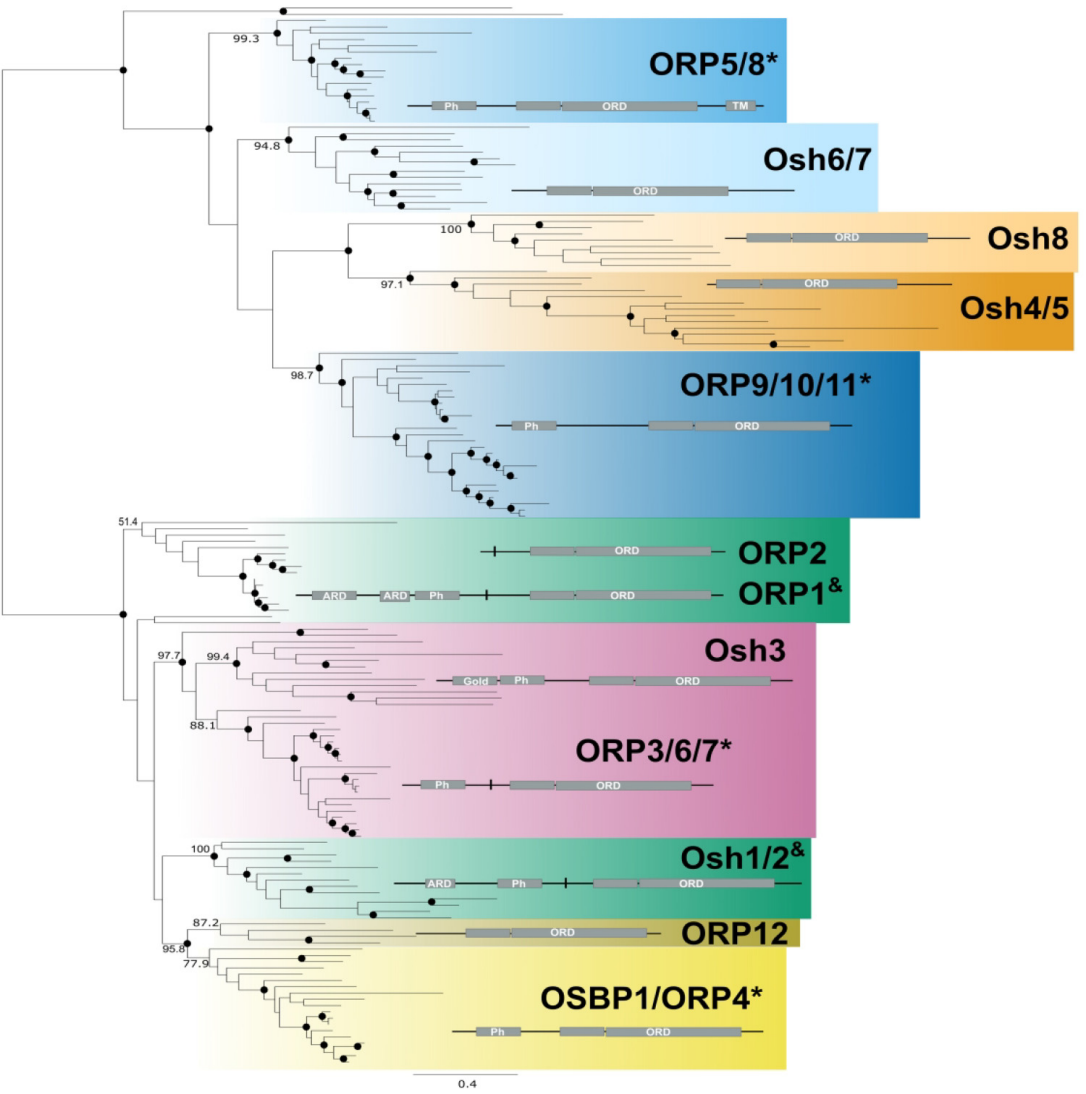
The ancestor of animals and fungi contained three OSBPs. This schematic tree is based on Figure S4. Nodes with bootstrap values greater than 90 are shown in a solid circle (•), and the bootstrap values that supported the grouping of the 11 major clades are shown on the branches leading to the node. The schematic protein structures of each homolog are shown. Asterisk denotes OSBPs with PH and ORD domains. Ampersand denotes OSBPs with ARD, PH, and ORD domains.

The *H. sapiens* genome encodes 12 OSBPs that facilitate lipid transport within the cell (Lehto et al., 2001). Like in fungi, the origin of these paralogues is unclear. We, therefore, reconstructed the phylogeny of holozoan ORP proteins to determine their evolutionary relationships (Figure 2 and S3). Our data show that the twelve human OSBPs group into five well-supported clades: ORP1/2, ORP3/6/7, OSBP1/ORP4, ORP5/8, and ORP9/10/11. Each of these clades includes single-celled protist species closely related to animals and therefore can be inferred to have been present in the ancestral holozoan (though orthologues from holozoan protists did not branch with ORP1/2, the presence of Ankyrin domains strongly suggests that they are related to ORP1/2 (Figure 3 and S4). In addition, we identified an ancient OSBP clade not present in any sampled vertebrate genome, which we named ORP12, that branches sister OSBP1/ORP4 with moderate support (Figure 2 and S3). We infer that the ancestral animal contained six OSBPs, one of which (ORP12) was lost in the lineage leading to vertebrates. The presence of six ancestral holozoan OSBPs suggests that a complex non-vesicular lipid trafficking pathway existed in the ancestor of animals. Five of the ancestral OSBPs likely functioned similarly to a subset of those found in *H. sapiens*, and ORP12 would have added to the complexity in a similar way to how additional paralogues in vertebrates add further complexity and nuance to the cell biology of non-vesicular lipid transfer.

### The Common Ancestor of Animals and Fungi had Three OSBPs

To determine if OSBP paralogues in *H. sapiens* and *S. cerevisiae* predate the diversification of animals and fungi, we reconstructed the phylogeny of opisthokont OSBPs (Figure 3 and S4). Since the ancestral holomycotan and holozoan had 5 and 6 OSBPs, respectively, we hypothesized that at least some of this complexity arose prior to the divergence of animals and fungi. While each clade from the holomycotan and holozoan trees was reconstructed with high support (i.e., ORP1/2, OSBP1/ORP4-ORP12, ORP5/8, ORP9/10/11, Osh1/2, Osh4/5-Osh8, and Osh6/7), only Osh3 and ORP3/6/7 came together to form a robustly supported ancient clade (Figure 3 and S4). Thus, to our surprise, our hypothesis was only partly validated—instead of a complex opisthokont ancestor, most of the complexity of animal and fungal non-vesicular membrane trafficking arose from independent duplications of OSBPs.

Even though no monophyletic groups (except Osh3-ORP3/6/7) uniting OSBP representatives from both animals and fungi were formed in the Opisthokont tree, structural and functional information can be used in combination with phylogenies to infer orthology. For example, in addition to Osh3 and ORP3/6/7 branching sister to one another, they also have similar structures and functions. Fungal Osh3 proteins have a similar domain organization to ORP3/6/7 as they all contain an FFAT motif, a PH domain, and an ORD (Lehto et al., 2004; Loewen et al., 2003). The ORD of ORP3 and Osh3 lack sterol binding due to it having a narrow ligand binding domain (Tong et al., 2013, 2021). In yeast, Osh3 regulates lipid metabolism and vesicular trafficking at ER-PM contact sites (Loewen et al., 2003; Roy & Levine, 2004; Schulz et al., 2009; Stefan et al., 2011). In humans, ORP3 similarly localizes near ER-PM contact sites and both Osh3 and ORP3 can trap PIPs including PI(4)P (Gulyas et al., 2020; Stefan et al., 2011; Yu et al., 2004). ORP6 likely functions similarly to ORP3 as it also localizes to ER-PM contact sites and downregulates PI(4)P levels (Mochizuki et al., 2018). ORP7 function remains somewhat unclear (Zhong et al., 2011). Thus, the similar domain structures, functions, and localizations of Osh3 in fungi and ORP3/6/7 in animals corroborate their orthology.

To extend our phylogenetic inferences, we investigated the domain organization and functions of other opisthokont OSBPs. Similar to fungal Osh1 and Osh2, animal ORP1 contains an ORD, FFAT motif, an ARD, and a PH domain (Lehto et al., 2001; Loewen et al., 2003). Through its ARD, ORP1 can bind to late endosomes and lysosomes (Johansson et al., 2005). Similarly, Osh1 uses its ARD to localize at nuclear-vacuole (aka lysosome) junctions (Kvam & Goldfarb, 2004, 2006; Levine & Munro, 2001). Found only in vertebrates, ORP2 is truncated, with only an ORD and facilitates lipid transfer at several MCSs (Kentala et al., 2015; Wang et al., 2019; Weber-Boyvat et al., 2015). ORP2 performs lipid transfer between PM and endosomes (Koponen et al., 2019; Wang et al., 2019) as well as at ER-lipid droplet MCSs (Hynynen et al., 2009), and promotes bidirectional exchange of cholesterol/PI(4,5)P_2_ between late and recycling endosomes (Takahashi et al., 2021). Similarly, Osh2 can be found at ER-PM MCSs and using their PH domain target PI(4)P or PI(4,5)P_2_ (Loewen et al., 2003; Maeda et al., 2013; Roy & Levine, 2004; Schulz et al., 2009; Stefan et al., 2011). Thus, ORP1 and Osh1/2 share a domain structure and have similar functions and localizations. We, therefore, suggest that the ancestral opisthokont contained a cholesterol-binding, ARD-containing, OSBP similar to ORP1/2 and Osh1/2.

**Figure 4.**
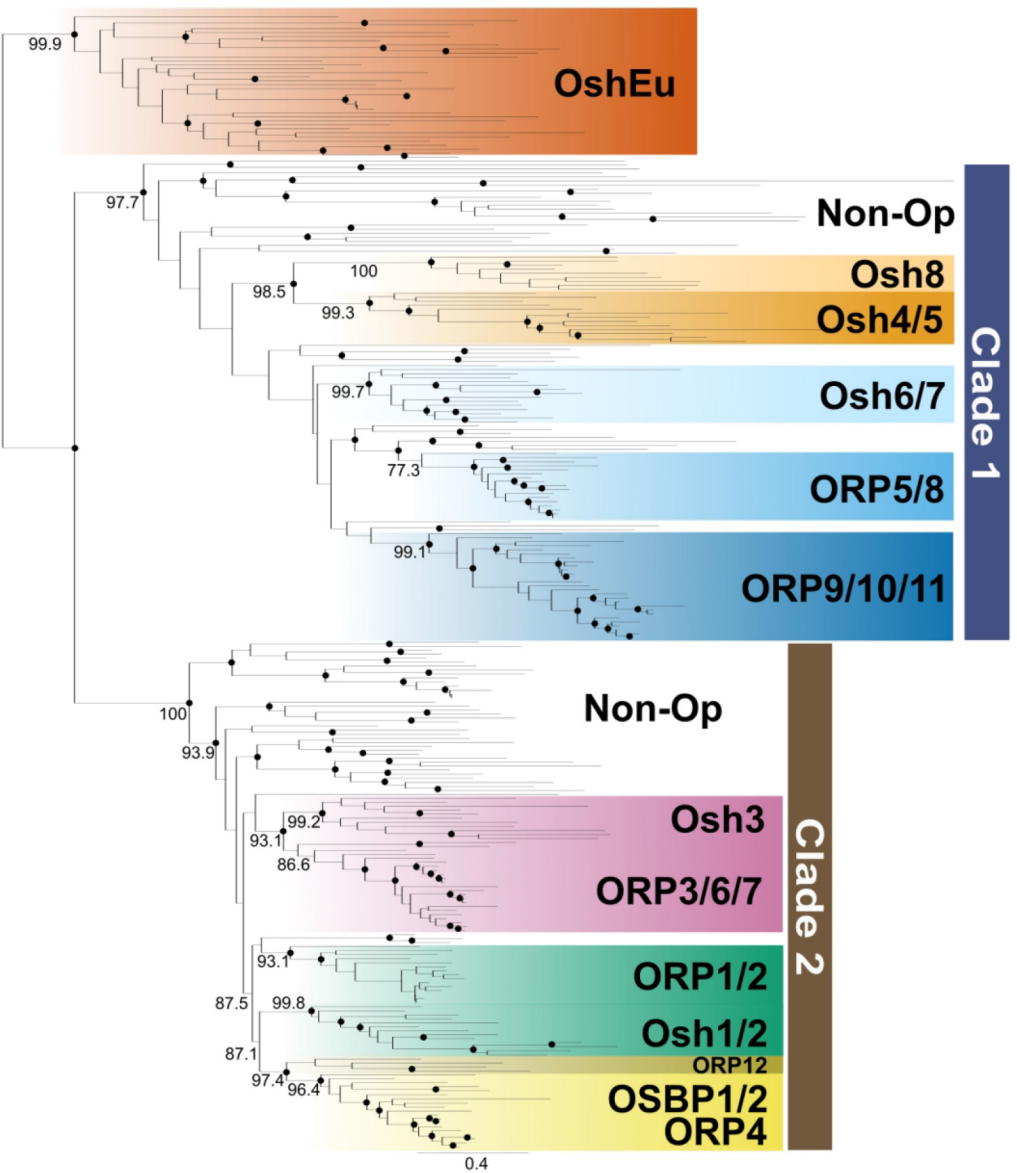
Three OSBPs were present in the ancestral eukaryote. This schematic tree is based on Figure S5. Sequences were collected from the EukProt dataset and NCBI. The tree indicates that there were three OSBPs in the last common ancestor of eukaryotes—OshEu, and one representative from Clade 1 and one from Clade 2. The OshEu clade was found in diverse protists, but not animals, fungi, or plants. Nodes with the bootstrap values greater than 90 are shown in a solid circle (•), and the bootstrap values supporting major clades are shown on the branches leading to the node.

The remaining orthologues retain fewer shared characteristics between animals and fungi. Although branching patterns are not indicative of clear orthology in the opisthokont tree (Figure 3 and S4), when the OSBP phylogeny is reconstructed using sequences from across eukaryotes (Figure 4 and S5) the situation becomes somewhat clearer. ORP5/8 and ORP9/10/11 branch sister to one another indicating that these proteins arose from an animal-specific duplication.

ORP5 and 8 and ORP9 both have PH domains suggesting that they share an evolutionary history. ORP5/8 additionally contains a C-terminal transmembrane domain which anchors the proteins to the ER membrane, whereas ORP9 contains a FFAT motif that enables VAP interactions at the ER. PH domains of ORP5/8 enable interaction with the PM (Lee & Fairn, 2018; Sohn et al., 2018). The ORD of ORP5/8 is similar to that of Osh6/7, and both have been shown to transport PS at ER-PM MCSs (Chung et al., 2015; Maeda et al., 2013). Osh6/7 contains only an ORD and are known to be cytosolic, but they can also localize at the ER-PM contact site via interaction with the ER-PM MCS protein Ist2 (Wong et al., 2021). Osh6/7 are also involved in stabilizing ER-PM membrane contact sites in yeast (Collado et al., 2019; Hoffmann et al., 2019; Manford et al., 2012). ORP5/8 have been recently reported to function at ER-mitochondria MCSs (Galmes et al., 2016), which may explain the lack of ER-mitochondria encounter structure (ERMES) in animals (Wideman et al., 2013).

While ORP5/8 and Osh6/7 appear to function similarly to one another at similar cellular locations (mitochondrial function notwithstanding), ORP9/10/11, though they appear to transport PS, localize differently. ORP9 mediates vesicular transport between the ER and the Golgi and regulates the sterol levels of the post-Golgi and endosomal compartments (Ngo & Ridgway, 2009). ORP10 lacks a FFAT motif but can heterodimerize with ORP9 and thereby interact with VAP to allow for PS/PI(4)P exchange at ER-Golgi and ER-endosome MCSs (Kawasaki et al., 2022; Nissilä et al., 2012; Zhou et al., 2010). ORP11 can bind sterols, PS, and PI(4)P (Maeda et al., 2013; Suchanek et al., 2007). In Figure 4 and S5, non-opisthokont OSBPs that branch at the base of Clade 1, appear to lack PH domains suggesting that the Osh6/7 domain structure is ancestral to the group and the PH domains characteristic of the holozoan paralogues were added in the animal expansions.

The remaining OSBP clades in opisthokonts likely represent lineage-specific duplications in animals and fungi. In animals, OSBP1 and ORP4 both contain a PH domain, FFAT motif, and an ORD and function similarly (Charman et al., 2014; Goto et al., 2012; Wyles et al., 2007). Both have the capacity to bind sterols or PI(4)P via the ORD, and can target PI(4)P using their PH domains. ORP4 can localize to the ER-Golgi contact site through its PH domain and can target PI(4)P. Through heterodimerization with OSBP they can regulate the PI(4)P levels in the Golgi (Pietrangelo & Ridgway, 2018). ORP12 is closely related to OSBP1/ORP4 but through domain analysis, it was found that ORP12 lacks a PH domain.

In fungi, Osh4/5 and Osh8 contain only an ORD. In *S. cerevisiae*, Osh4 regulates post-Golgi vesicles involved in polarized exocytosis (Delfosse et al., 2020). Due to there being a vast abundance of Osh4 and lipid exchange activity, Osh4 could transfer large amounts of sterols from the ER to the *trans-*Golgi enabling exocytic vesicles be loaded with sterols before interacting with the PM (Delfosse et al., 2020). Osh5 shares 70% identity with Osh4, but its function remains elusive (Beh et al., 2001). The function of Osh8 is unknown. However, if the function of Osh4/5 in polarized exocytosis is conserved in diverse fungi, and Osh8 and Osh4/5 come from an ancient fungal gene duplication, it is possible that Osh8 functions similarly. Since Osh8 was lost in the lineage leading to Saccharomycotina (compare Figure S1 to Figure 1), Osh8 is not required in the yeast lineage. This leads us to hypothesize that Osh8 may be involved in polarized exocytosis in filamentous growth. This could be tested in the model filamentous fungus *Neurospora crassa*. Since animal OSBP1/ORP4 and fungal Osh4 have similar functions at the Golgi, it is attractive to hypothesize that they may share an ancestral function, however, their branching in Clade 1 vs Clade 2 (Figure 4) makes this impossible, and instead any similarity is likely due to convergence.

### The Last Eukaryotic Common Ancestor (LECA) had Three OSBPs

To determine the OSBP complement of the ancestral eukaryote, we reconstructed the phylogeny of OSBPs including sequences from every major eukaryotic lineage. As detailed above, the opisthokont groups were faithfully reconstructed (Osh1/2, Osh3 and ORP3, Osh4/5+Osh8, Osh6/7, OSBP1/ORP4, ORP3/6/7, ORP5/8+ORP12, and ORP9/10/11). In addition, OSBP clades were recovered that include representatives from red algae, green algae, plants, protists, and amoebae. However, none of these branches strongly with any particular opisthokont OSBP orthologue (white branches in Figure 4), except a few amoebozoans that branch with ORP5/8—though this is likely due to long-branch attraction. Previous research on *Arabidopsis thaliana* identified 12 OSBPs (Umate, 2011). We included all of these in earlier iterations of Figure 4 but removed six as they branched with full support with the six *A. thaliana* OSBPs kept in the tree. Again, no *A. thaliana* OSBP branches with strong support with any opisthokont orthologue (Figure 4 and S5). Similarly, no non-opisthokont protist OSBP branched strongly with any opisthokont orthologue, except a few with ORP5/8 and Osh6/7, which we believe are due to long-branch attraction artifacts.

Surprisingly, an additional major clade (OshEu) was identified that lacks sequence representatives from both animals and fungi (though an OSBP from *Capsaspora owczarzaki*, an opisthokont protist, branches within this group). This clade represents a newly discovered eukaryotic OSBP orthologue that has persisted since the last eukaryotic common ancestor. Sequence inspection did not reveal any clearly conserved amino acids unique to OshEu suggesting that this clade may have formed due to long branch attractions (Bergsten, 2005). However, since OshEu is clearly excluded from Clade 1 and Clade 2, and representatives from each major eukaryote clade fall within all three, we speculate that the Last Eukaryote Common Ancestor contained three OSBPs, an OshEu orthologue of unknown function, one OSBP distantly related to Clade 1 (Osh8-Osh4/5-Osh6/7-ORP5/8-ORP9/10/11—top of Figure 4 and S5)—perhaps capable of transporting PS and PI4P, and one distantly related to Clade 2 (Osh1/2-ORP1/2-ORP12/OSBP1/ORP4-ORP3/6/7/Osh3)—perhaps capable of transporting sterol and PI4P. Since the essential function of OSBPs in yeast appears to be ONLY the transport of PI4P (Tong et al., 2021), and several eukaryotes contain only a single OSBP, it is tempting to speculate that the ancestral, perhaps even pre-LECA, function of the primordial OSBP was to facilitate retrograde transport of PI4P from the plasma membrane to the ER where it would be converted to PI. Later, secondary lipids were added as nuanced non-vesicular lipid transport functions expanded. Further functional characterization and deeper phylogenetic investigations (including OSBPs from more eukaryotes not yet sequenced) are required to validate these possibilities.

## Conclusions

The evolution of eukaryotic non-vesicular lipid transport is complicated. While non-vesicular lipid transport is an essential and ancient feature of eukaryotic cells, how it happens, and which proteins are required for lipid transport between which membranes varies considerably between different lineages. For example, while ERMES is nearly ubiquitous in the fungal lineage, it is absent in animals and spread patchily across other eukaryotic groups Wideman et al., 2013). Our work on OSBPs confirms the complex evolutionary history of non-vesicular lipid transport.

**Figure 5.**
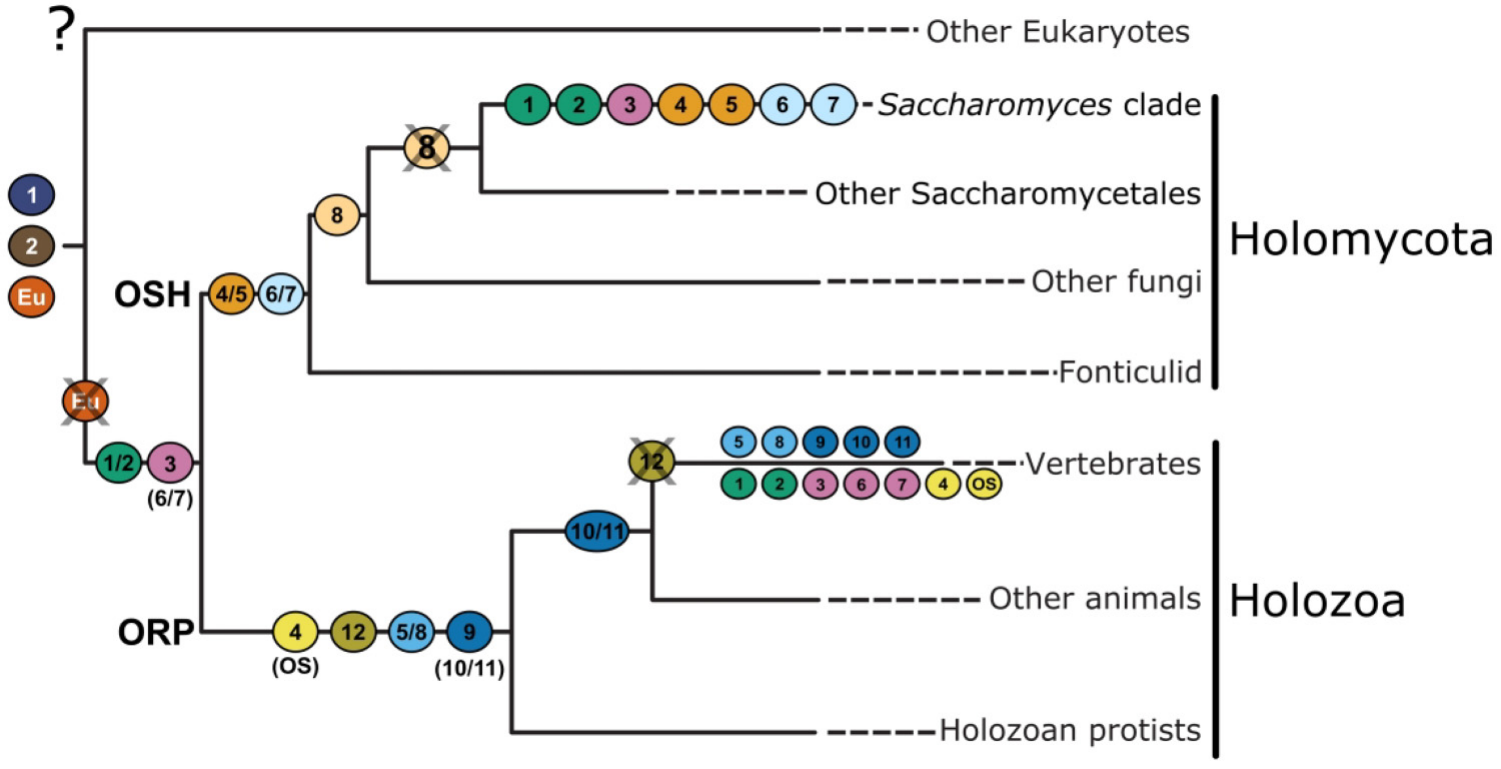
Schematic of OSBP duplications and losses in the Opisthokonts. The ancestral eukaryote had only three OSBPs (OshEu, and representatives from Clade 1 and Clade 2—see Figure 4). OshEu was lost prior to the diversification of animals and fungi. The ancestral opisthokont also had three OSBPs due to a duplication of the Clade 2 representative resulting in Osh1/2-like and Osh3-like ancestral OSBPs. From the ancestral opisthokont, animals and fungi followed independent trajectories of OSBP expansion. The ancestral fungus had five OSBPs (Osh1/2 and Osh3 as well as Osh4/5, Osh6/7, and Osh8—resulting from duplications of the ancestral Clade 1 homolog). Most fungi retain this set of five ancestral OSBPs. However, the Saccharomycotina lost Osh8. The ancestral animal had six OSBPs (ORP1/2, ORP3/6/7 in addition to ORP4/OSBP, ORP5/8, ORP9/10/11, and ORP12 all resulting from lineage-specific duplications). Vertebrates lost ORP12. The whole genome duplication in the lineage leading to *S. cerevisiae* led to its seven OSBPs. Duplications in the vertebrate lineage led to the 12 OSBPs in humans.

The ancestral eukaryote had at most three OSBPs (Figure 5). One of these (OshEu) was lost from animals and fungi. In the lineage leading to animals and fungi, the two remaining OSBPs had N-terminal PH domains and one experienced a gene duplication event. Ankyrin repeats were added to the N-terminus of one of the duplicates. Thus, three OSBPs were present in the ancestor of animals and fungi, two consisted of a PH domain followed by an ORD (representing the ancestors of and ORP3/6/7–Osh3 and ORP5/8/9/10/11–Osh4/5/6/7/8) and another had ankyrin repeats, a PH domain, and an ORD (representing the ancestor of ORP1/2–Osh1/2) (Figure 5). From here, independent duplications occurred in the lineages leading to animals and fungi.

In the lineage leading to fungi, a duplication resulted in Osh4/5 and Osh6/7. After the divergence of fonticulids, Osh4/5 further duplicated, resulting in the five ancestral fungal Osh proteins (Osh1/2, Osh3, Osh4/5, Osh6/7, and Osh8). In the Saccharomycetales, Osh8 was lost. In the lineage leading to *S. cerevisiae*, a whole genome duplication resulted in the seven Osh proteins seen in this species. In the lineage leading to animals, similar gene duplications occurred. Even prior to the origin of holozoans four duplications occurred resulting in six ancestral ORPs: ORP1/2, ORP3, ORP4/OSBP, ORP12, ORP5/8, and ORP9/10/11. Early in the evolution of animals, another duplication occurred resulting in ORP9 and ORP10/11. The loss of ORP12 occurred repeatedly in several holozoan groups. Finally, several duplications occurred prior to the divergence of vertebrates resulting in the plethora of OSBPs present in this lineage.

In conclusion, our phylogenetic analyses show that a complex lipid transport system was present in the ancestor of fungi (five OSBPs), and animals (six OSBPs). Why this complexity arose from their simpler predecessors, which only had three OSBPs, is unclear. Why animals and fungi require more complexity in their non-vesicular lipid transport systems than other eukaryotes remains an open question. Perhaps certain lineages of eukaryotes underwent expansions of other lipid transport proteins, or perhaps their vesicular transport systems expanded in other ways. In addition to the known complexity in animals and fungi, we identified Osh8, ORP12, and OshEu, three ancient orthologues not found in *H. sapiens* or *S. cerevisiae*. Further functional investigations of OSBPs in *H. sapiens* and *S. cerevisiae* are required to pin down the exact roles each OSBP plays in these organisms. However, to gain a full picture of how non-vesicular lipid trafficking works in eukaryotes, other model systems must be investigated.

## Supplemental Material

sj-fa-1-ctc-10.1177_25152564221150428 - Supplemental material for Evolutionary History of Oxysterol-Binding Proteins Reveals Complex History of Duplication and Loss in Animals and Fungi Click here for additional data file. Supplemental material, sj-fa-1-ctc-10.1177_25152564221150428 for Evolutionary History of Oxysterol-Binding Proteins Reveals Complex History of Duplication and Loss in Animals and Fungi by Rohan P. Singh, Yu-Ping Poh, Savar D. Sinha and Jeremy G. Wideman in Contact

sj-docx-2-ctc-10.1177_25152564221150428 - Supplemental material for Evolutionary History of Oxysterol-Binding Proteins Reveals Complex History of Duplication and Loss in Animals and Fungi Click here for additional data file. Supplemental material, sj-docx-2-ctc-10.1177_25152564221150428 for Evolutionary History of Oxysterol-Binding Proteins Reveals Complex History of Duplication and Loss in Animals and Fungi by Rohan P. Singh, Yu-Ping Poh, Savar D. Sinha and Jeremy G. Wideman in Contact

sj-pdf-3-ctc-10.1177_25152564221150428 - Supplemental material for Evolutionary History of Oxysterol-Binding Proteins Reveals Complex History of Duplication and Loss in Animals and Fungi Click here for additional data file. Supplemental material, sj-pdf-3-ctc-10.1177_25152564221150428 for Evolutionary History of Oxysterol-Binding Proteins Reveals Complex History of Duplication and Loss in Animals and Fungi by Rohan P. Singh, Yu-Ping Poh, Savar D. Sinha and Jeremy G. Wideman in Contact

sj-pdf-4-ctc-10.1177_25152564221150428 - Supplemental material for Evolutionary History of Oxysterol-Binding Proteins Reveals Complex History of Duplication and Loss in Animals and Fungi Click here for additional data file. Supplemental material, sj-pdf-4-ctc-10.1177_25152564221150428 for Evolutionary History of Oxysterol-Binding Proteins Reveals Complex History of Duplication and Loss in Animals and Fungi by Rohan P. Singh, Yu-Ping Poh, Savar D. Sinha and Jeremy G. Wideman in Contact

sj-pdf-5-ctc-10.1177_25152564221150428 - Supplemental material for Evolutionary History of Oxysterol-Binding Proteins Reveals Complex History of Duplication and Loss in Animals and Fungi Click here for additional data file. Supplemental material, sj-pdf-5-ctc-10.1177_25152564221150428 for Evolutionary History of Oxysterol-Binding Proteins Reveals Complex History of Duplication and Loss in Animals and Fungi by Rohan P. Singh, Yu-Ping Poh, Savar D. Sinha and Jeremy G. Wideman in Contact

sj-pdf-6-ctc-10.1177_25152564221150428 - Supplemental material for Evolutionary History of Oxysterol-Binding Proteins Reveals Complex History of Duplication and Loss in Animals and Fungi Click here for additional data file. Supplemental material, sj-pdf-6-ctc-10.1177_25152564221150428 for Evolutionary History of Oxysterol-Binding Proteins Reveals Complex History of Duplication and Loss in Animals and Fungi by Rohan P. Singh, Yu-Ping Poh, Savar D. Sinha and Jeremy G. Wideman in Contact

sj-pdf-7-ctc-10.1177_25152564221150428 - Supplemental material for Evolutionary History of Oxysterol-Binding Proteins Reveals Complex History of Duplication and Loss in Animals and Fungi Click here for additional data file. Supplemental material, sj-pdf-7-ctc-10.1177_25152564221150428 for Evolutionary History of Oxysterol-Binding Proteins Reveals Complex History of Duplication and Loss in Animals and Fungi by Rohan P. Singh, Yu-Ping Poh, Savar D. Sinha and Jeremy G. Wideman in Contact
