# Supplementary material for: Evolutionary History of Oxysterol-Binding Proteins Reveals Complex History of Duplication and Loss in Animals and Fungi
Source: Contact (Thousand Oaks). 2023 Jan 11;6:25152564221150428. doi: 10.1177/25152564221150428 (PMC10243569; doi:10.1177/25152564221150428)
Supplement: sj-docx-2-ctc-10.1177_25152564221150428 - Supplemental material for Evolutionary History of Oxysterol-Binding Proteins Reveals Complex History of Duplication and Loss in Animals and Fungi [file sj-docx-2-ctc-10.1177_25152564221150428.docx]

**Figure S1.** **Four OSBPs were present in the ancestor of Saccharomycotina.** The detailed phylogenetic tree of Saccharomycotina was constructed using sequences collected from the JGI mycocosm database. The tree indicates that there were four Osh proteins found in the last common ancestor of Saccharomycotina (Osh1/Osh2, Osh3, Osh4/Osh5, and Osh6/Osh7. This tree was reconstructed using the LG+R8 model. Bootstraps values greater than 80 are as indicated.

**Figure S2.** The detailed phylogenetic tree of the OSBP proteins in Holomycota. This figure is the original phylogenetic tree of Figure 1 with detailed accession numbers and species names of each taxa. The OSBPs of *S. cerevisiae* are highlighted in bold.

**Figure S3.** The detailed phylogenetic tree of the OSBP proteins in Holozoa. This figure is the original phylogenetic tree of Figure 2 with detailed accession numbers and species names of each taxon. The OSBPs of *H. sapiens* are highlighted in bold.

**Figure S4.** The detailed phylogenetic tree of the OSBP proteins in Opisthokonta. This figure is the original phylogenetic tree of Figure 3 with detailed accession numbers and species names of each taxon. The OSBPs of *S. cerevisiae* and *H. sapiens* are highlighted in bold.

**Figure S5.** The detailed phylogenetic tree of the OSBP proteins in Eukaryota. This figure is the original phylogenetic tree of Figure 4 with detailed accession numbers and species names of each taxon. The OSBPs of *S. cerevisiae* and *H. sapiens* are highlighted in bold.
