# Supplementary figures and images for: Evolutionary History of Oxysterol-Binding Proteins Reveals Complex History of Duplication and Loss in Animals and Fungi
Source: Contact (Thousand Oaks). 2023 Jan 11;6:25152564221150428. doi: 10.1177/25152564221150428 (PMC10243569; doi:10.1177/25152564221150428)

Figure S1

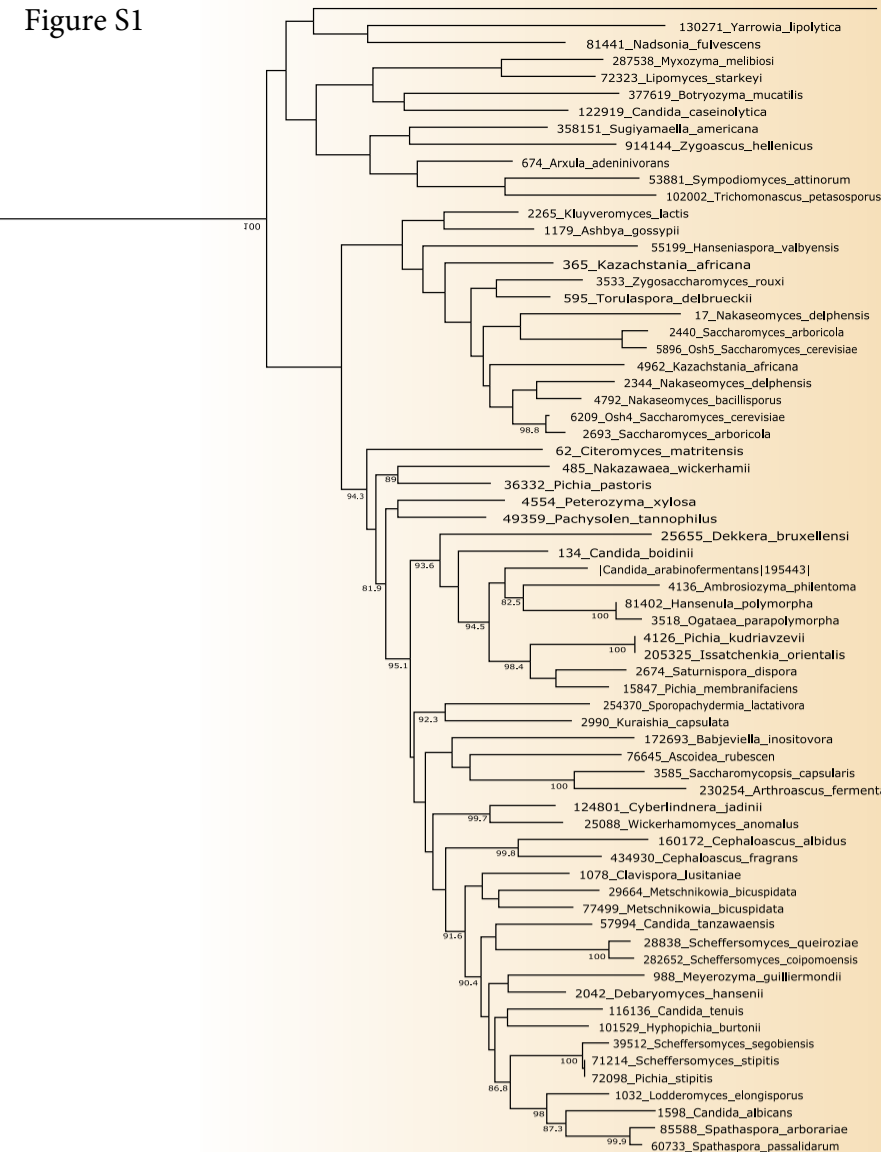

# Osh4/5

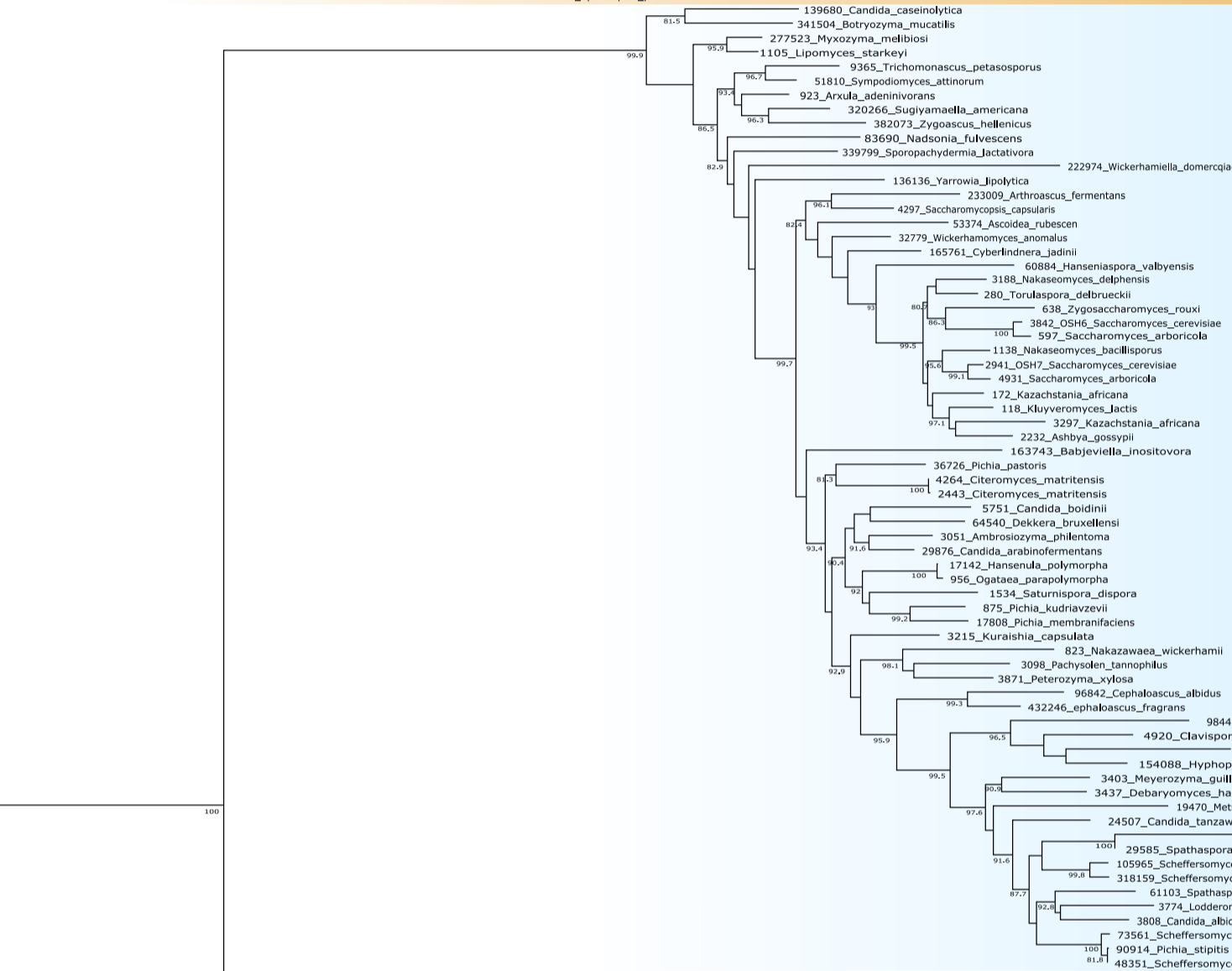

# Osh6/7

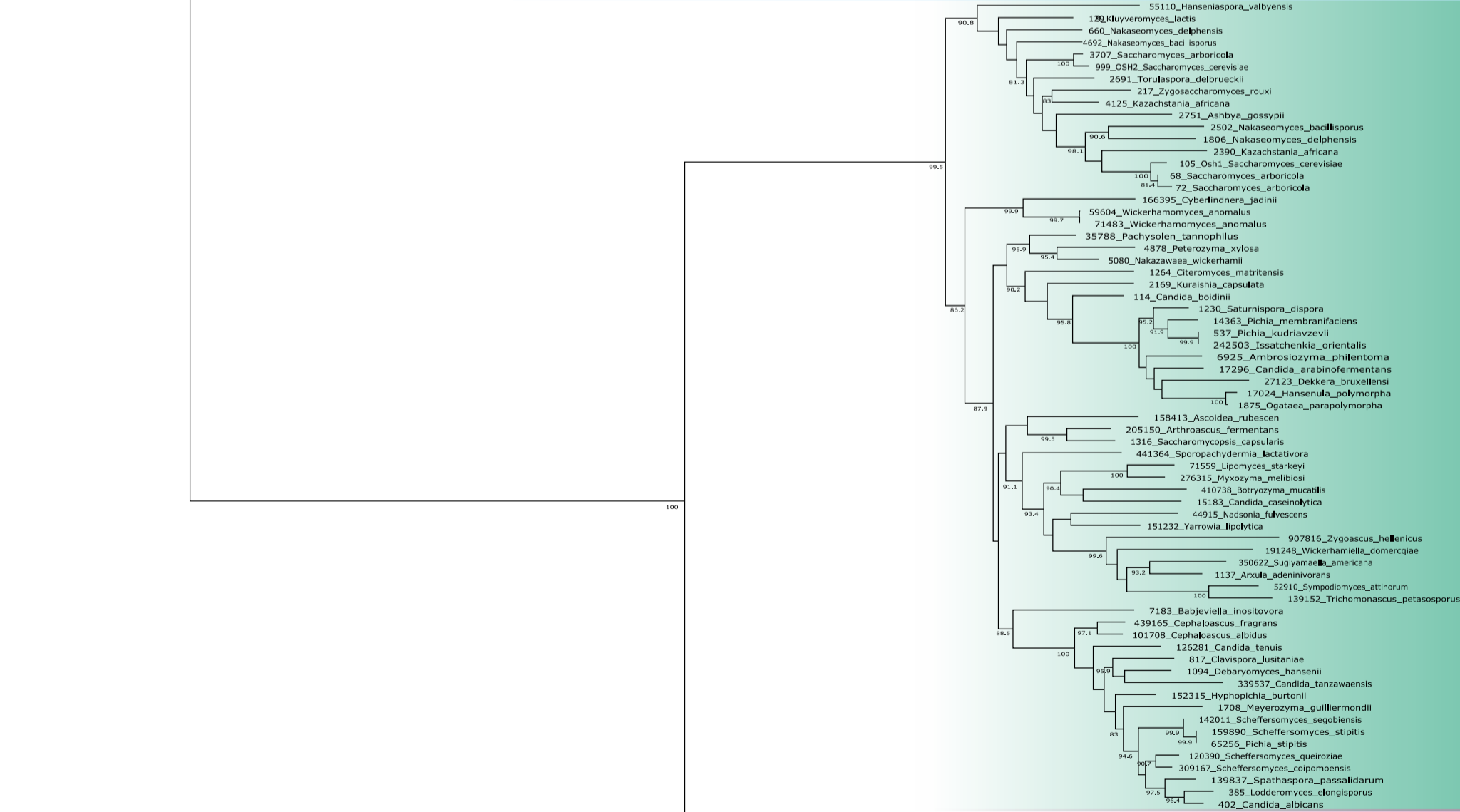

# Osh1/2

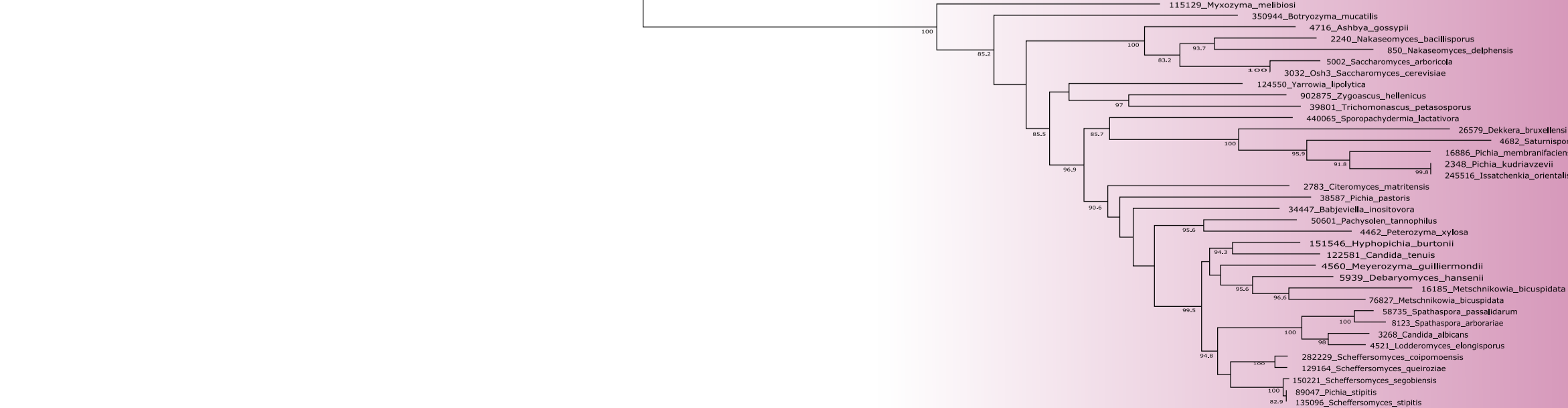

# Osh3

Supplement: sj-pdf-3-ctc-10.1177_25152564221150428 - Supplemental material for Evolutionary History of Oxysterol-Binding Proteins Reveals Complex History of Duplication and Loss in Animals and Fungi [file sj-pdf-3-ctc-10.1177_25152564221150428.pdf]

Figure S2

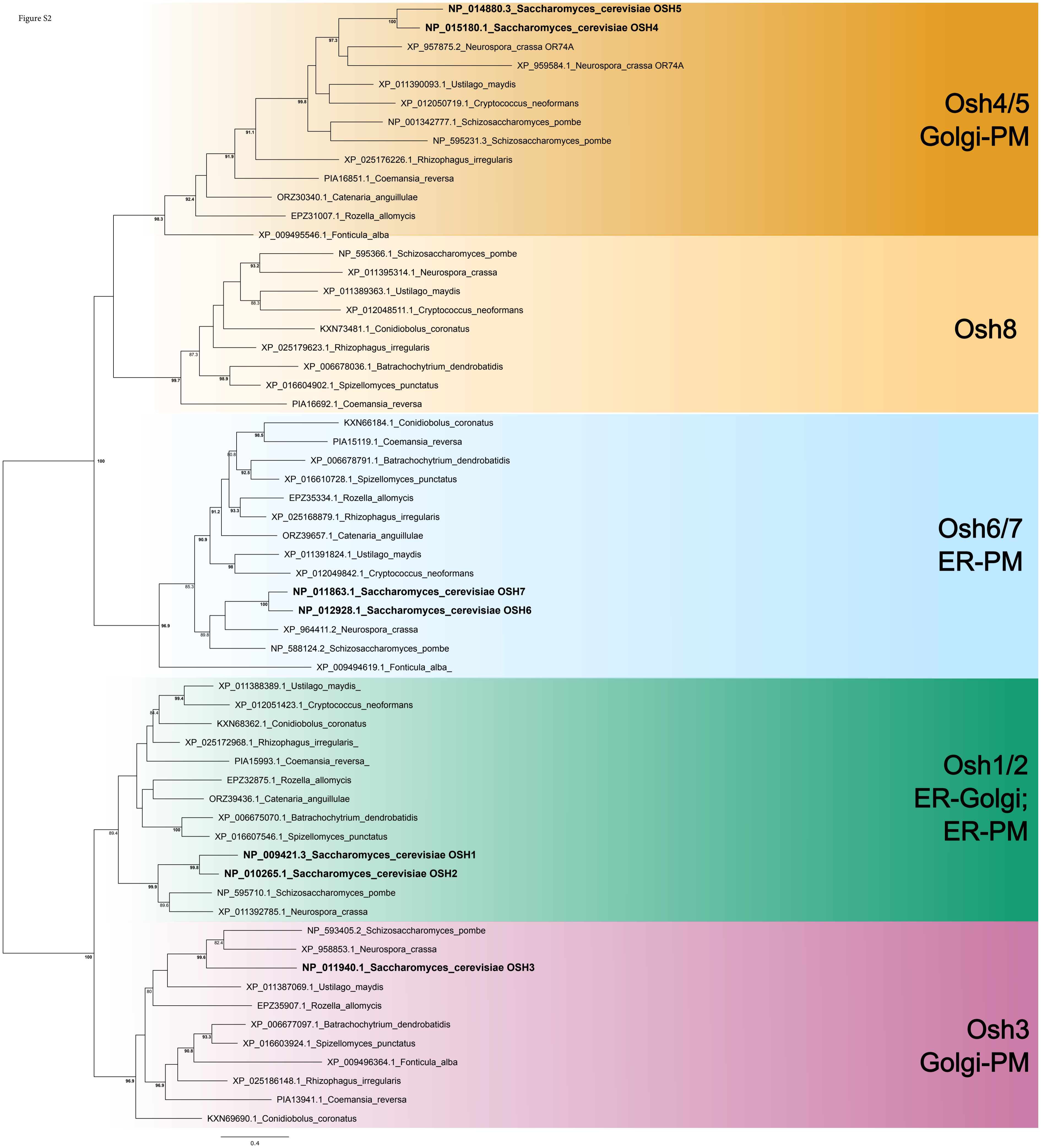

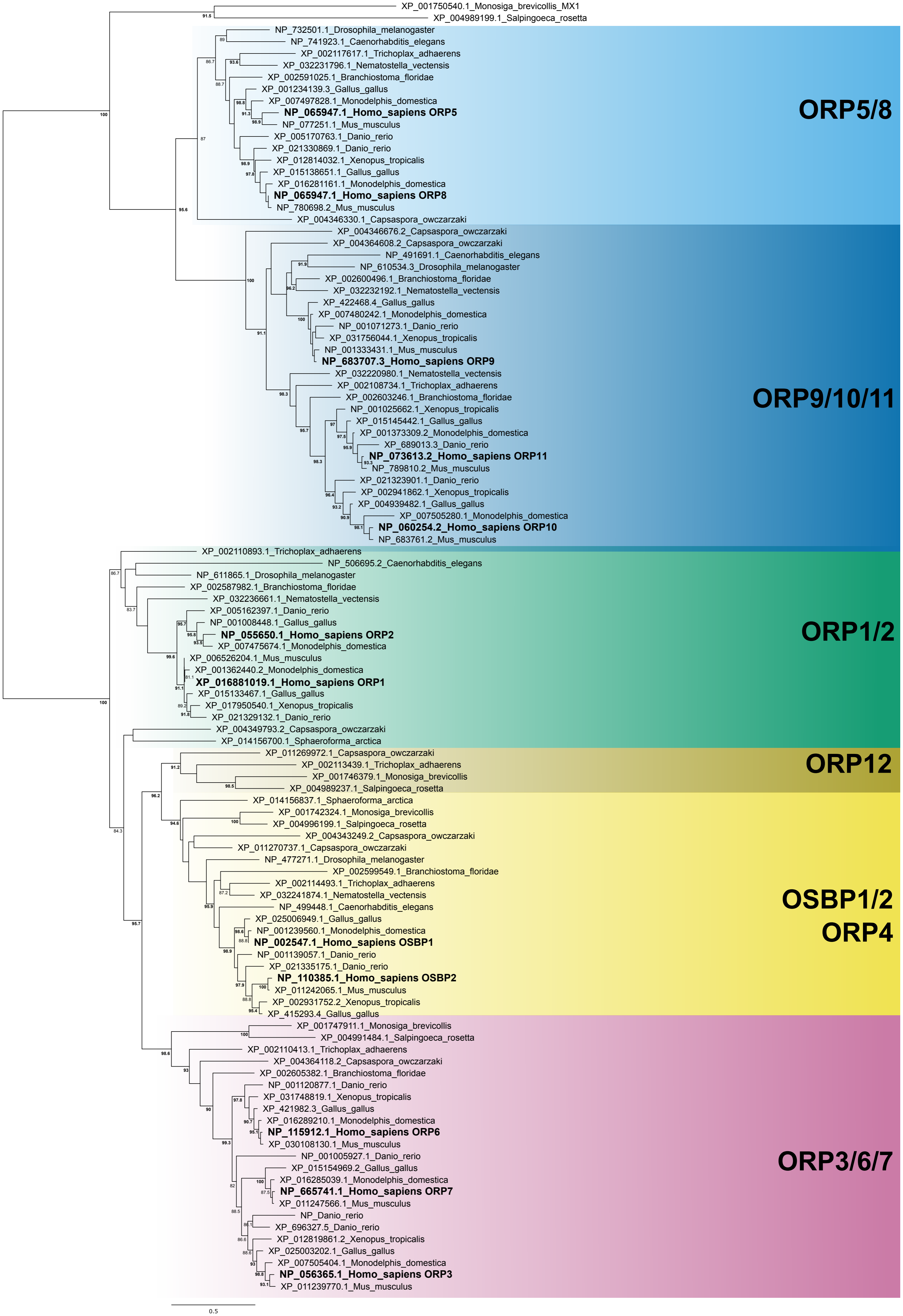

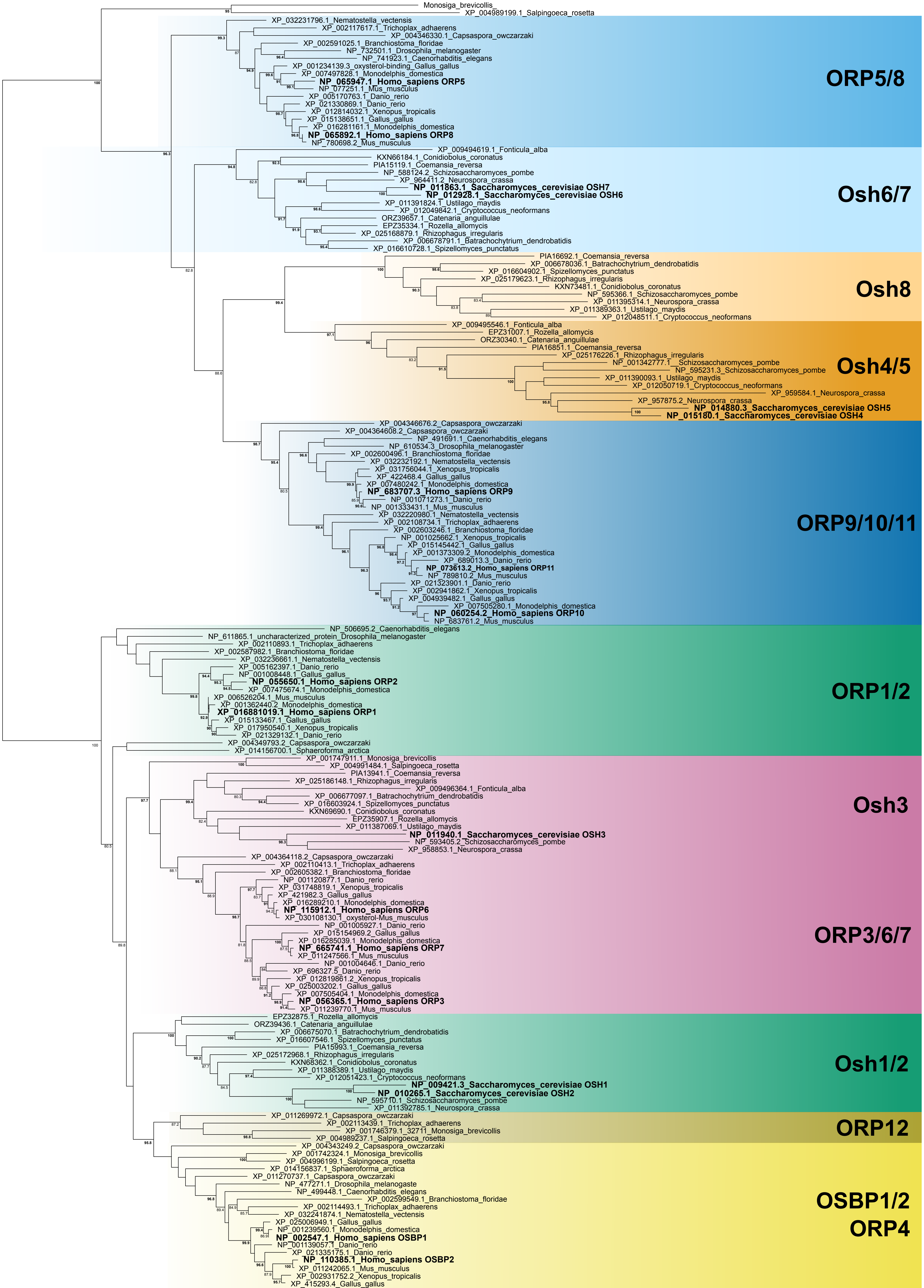

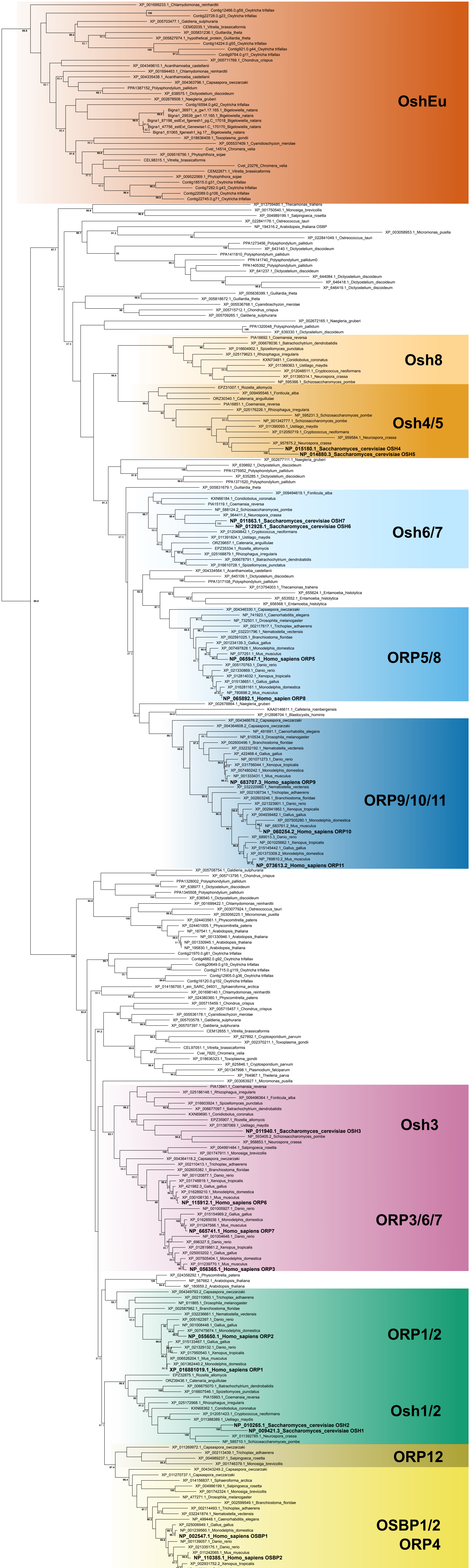

OshEu

Osh8

Osh4/5

Osh6/7

ORP5/8

ORP9/10/11

Osh3

ORP3/6/7

ORP1/2

Osh1/2

ORP12

OSBP1/2

ORP4

Supplement: sj-pdf-4-ctc-10.1177_25152564221150428 - Supplemental material for Evolutionary History of Oxysterol-Binding Proteins Reveals Complex History of Duplication and Loss in Animals and Fungi [file sj-pdf-4-ctc-10.1177_25152564221150428.pdf]

Figure S3

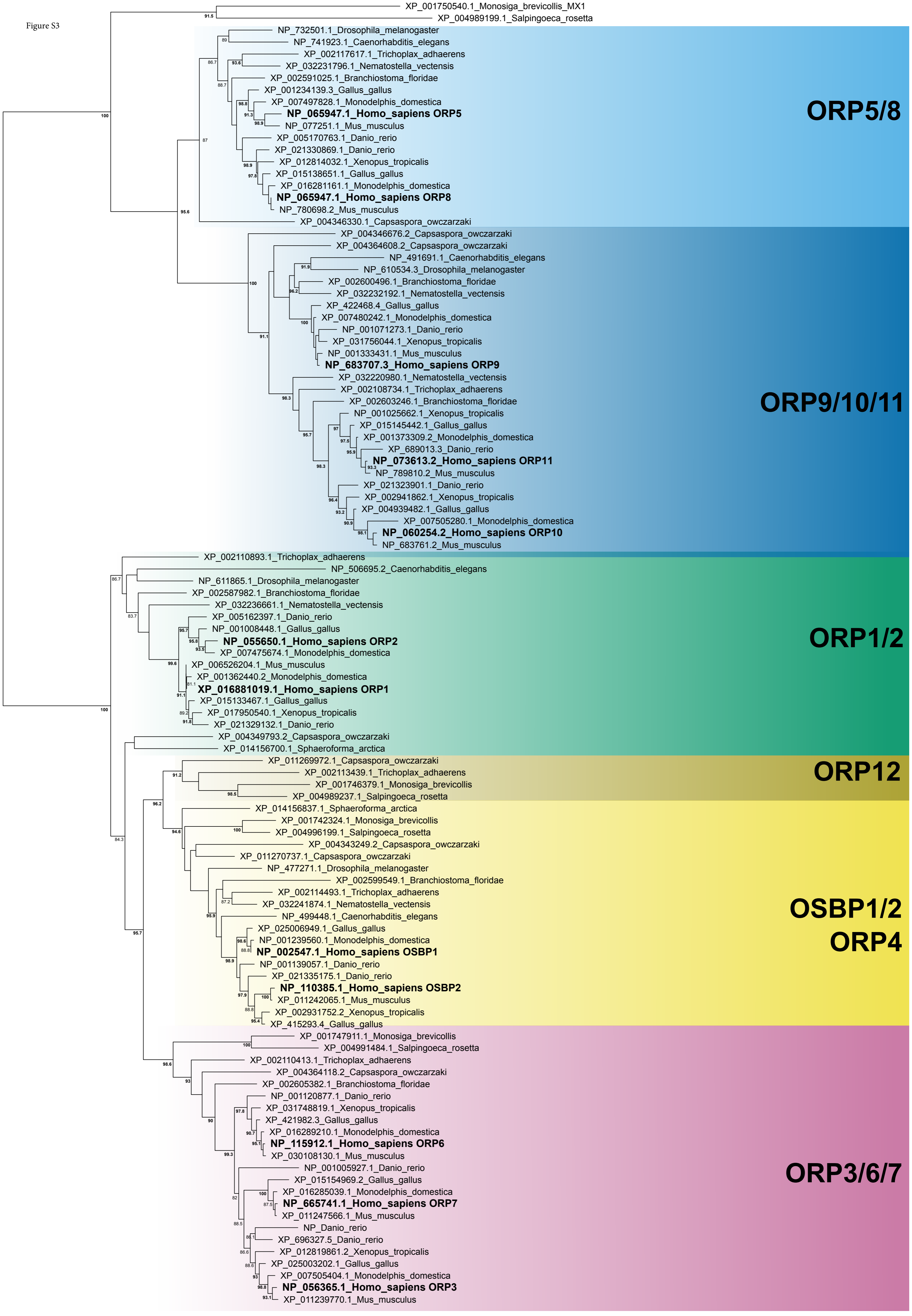

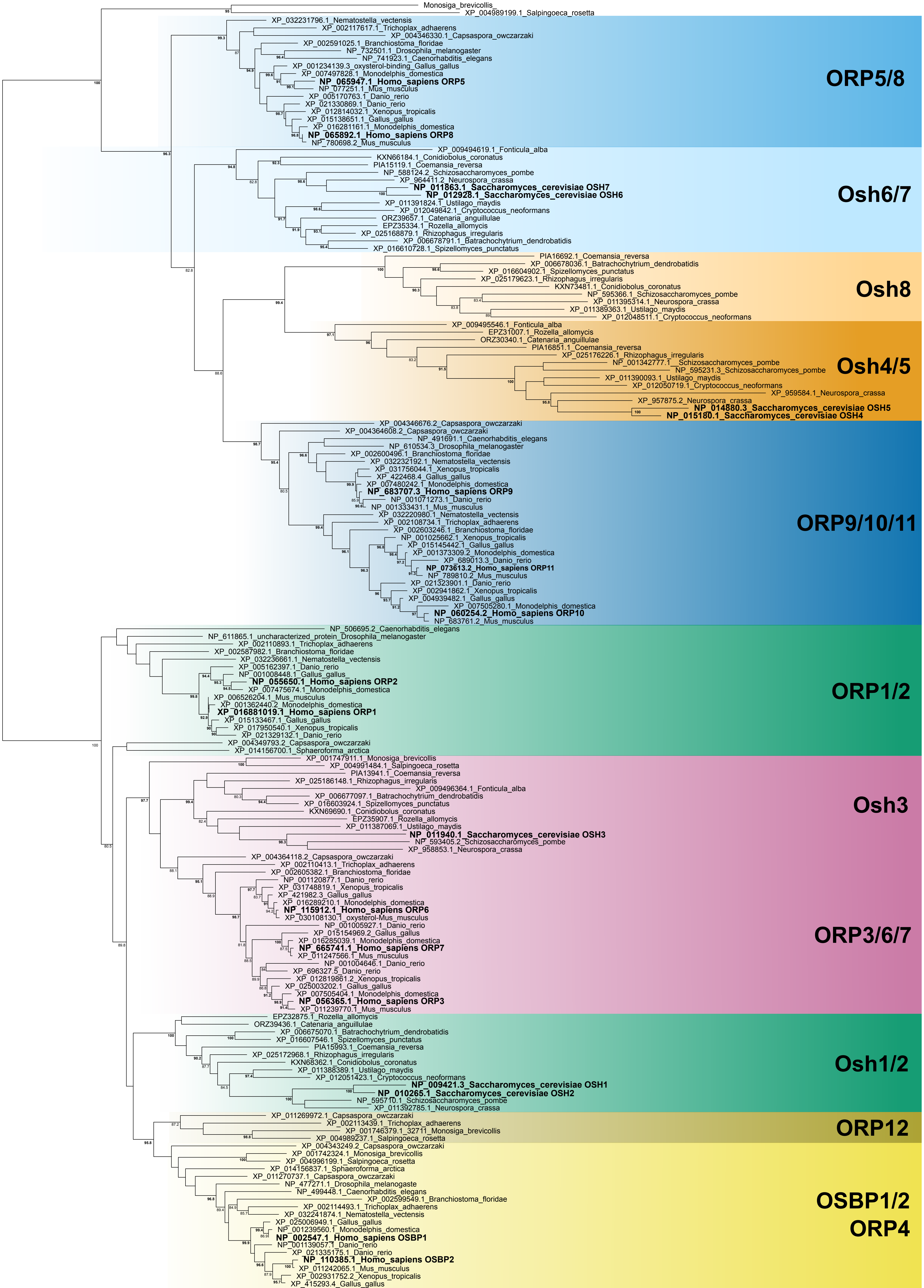

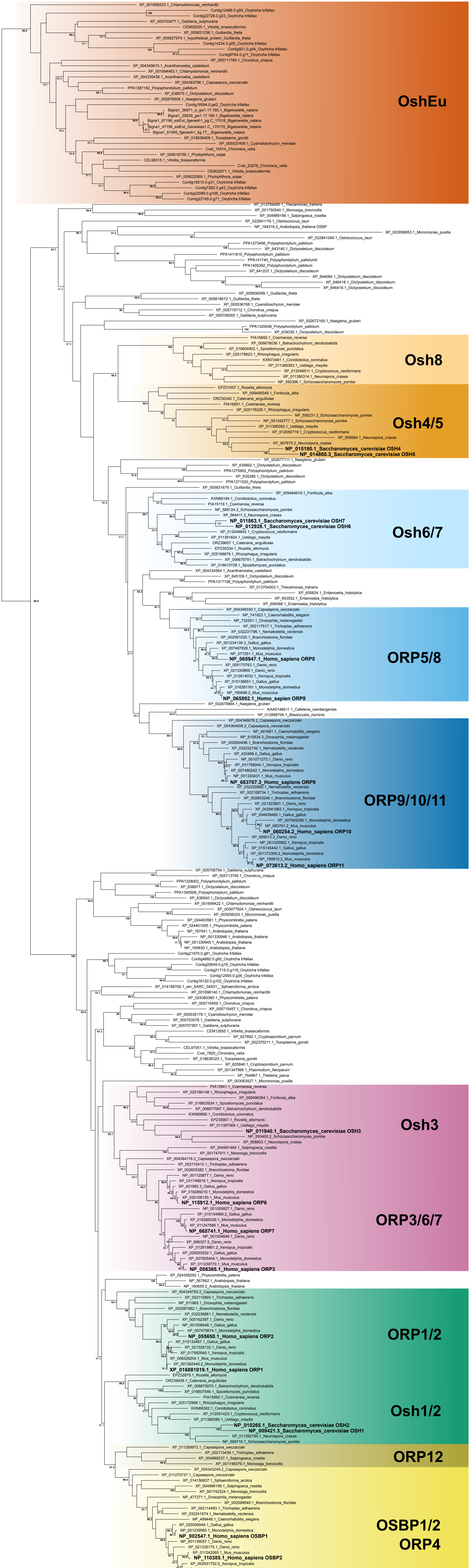

OshEu

Osh8

Osh4/5

Osh6/7

ORP5/8

ORP9/10/11

Osh3

ORP3/6/7

ORP1/2

Osh1/2

ORP12

OSBP1/2

ORP4

Supplement: sj-pdf-5-ctc-10.1177_25152564221150428 - Supplemental material for Evolutionary History of Oxysterol-Binding Proteins Reveals Complex History of Duplication and Loss in Animals and Fungi [file sj-pdf-5-ctc-10.1177_25152564221150428.pdf]

Figure S4

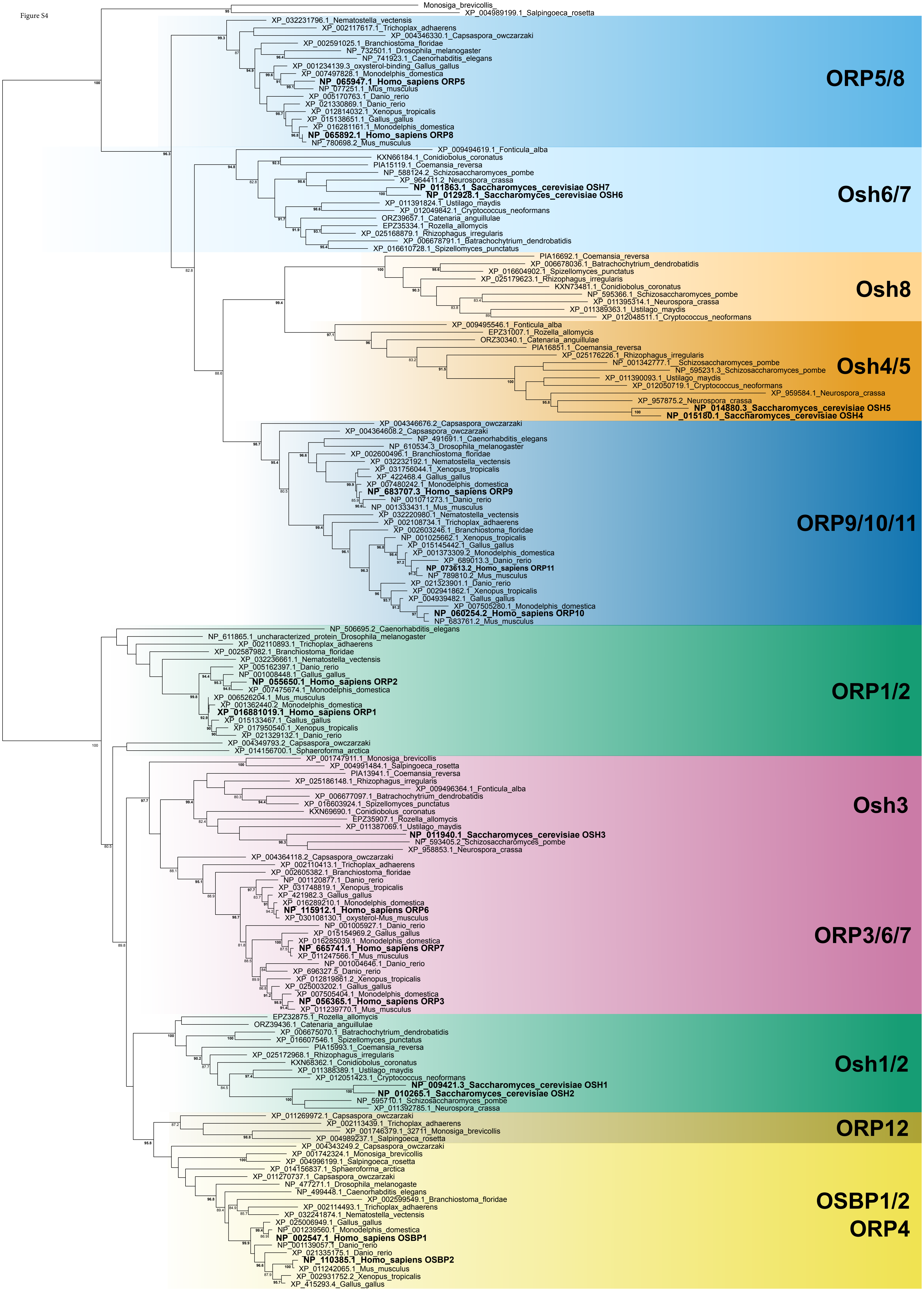

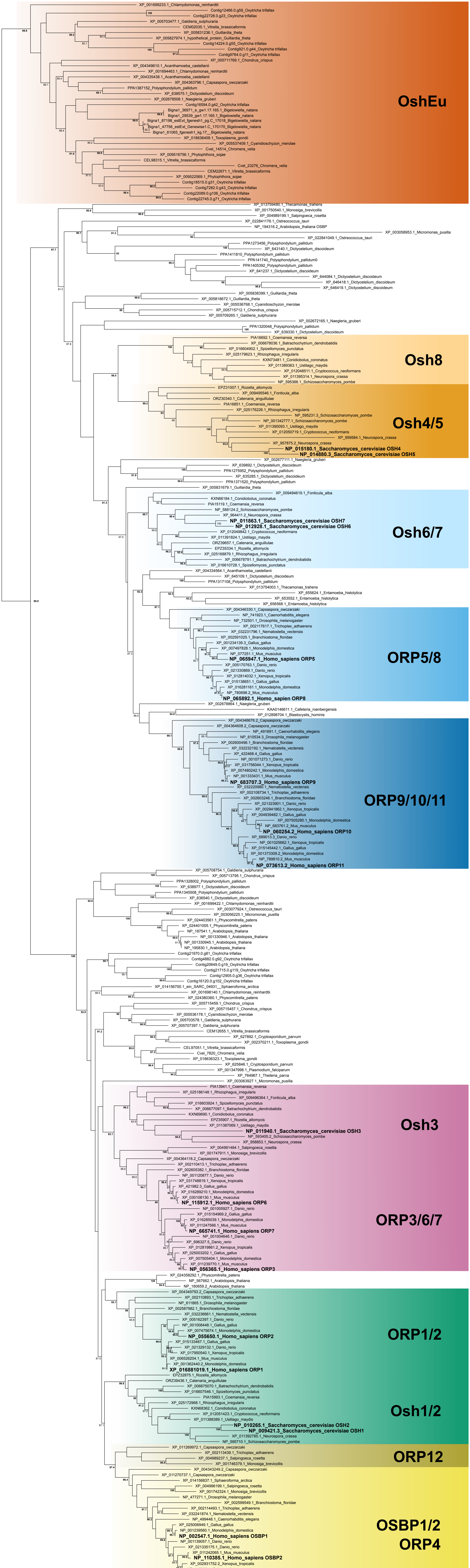

Supplement: sj-pdf-6-ctc-10.1177_25152564221150428 - Supplemental material for Evolutionary History of Oxysterol-Binding Proteins Reveals Complex History of Duplication and Loss in Animals and Fungi [file sj-pdf-6-ctc-10.1177_25152564221150428.pdf]

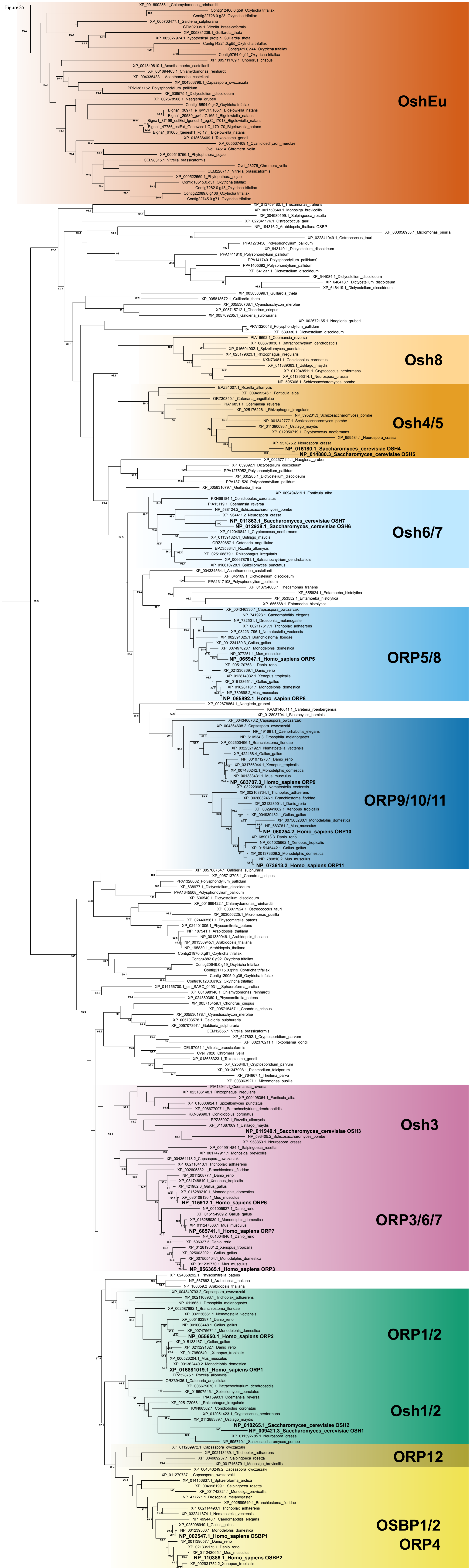

Supplement: sj-pdf-7-ctc-10.1177_25152564221150428 - Supplemental material for Evolutionary History of Oxysterol-Binding Proteins Reveals Complex History of Duplication and Loss in Animals and Fungi [file sj-pdf-7-ctc-10.1177_25152564221150428.pdf]
